# Modulation of Chemokine- and Adhesion-Molecule Gene Expression and Recruitment of Neutrophil Granulocytes in Rat and Mouse Liver after a Single Gadolinium Chloride or Zymosan Treatment

**DOI:** 10.3390/ijms19123891

**Published:** 2018-12-05

**Authors:** Shakil Ahmad, Giuliano Ramadori, Federico Moriconi

**Affiliations:** 1 Department of Gastroenterology and Endocrinology, University Hospital, Georg-August University Goettingen, 37075 Goettingen, Germany; giulianoramadori@gmail.com (G.R.); federicomoriconi@hotmail.it (F.M.); 2 Department of Cardiology and Pneumology, University Hospital, Georg-August University Goettingen, 37075 Goettingen, Germany; 3 GastroCentro, Via Trevano 38, 6900 Lugano, Switzerland

**Keywords:** inflammation, Kupffer cells, macrophages, chemokine, phagocytosis

## Abstract

Kupffer cells are professional phagocytes of the liver clearing bacteria from portal blood. Their clearance capacity, however, can be overwhelmed, transforming them into critical mediators of hepatic-injury. We investigated the consequences of selective Kupffer cell-overload by intraperitoneally administering pyrogen-free gadolinium chloride (GdCl_3_) or Zymosan into rats and into endotoxin-resistant mice (C3H/HeJ). The number of myeloperoxidase-positive (MPO^+^) cells increased at 3 h mainly around the portal vessel after both GdCl_3_ and Zymosan treatment. Simultaneously, GdCl_3_ administration reduced detectability of ED-1^+^ (but not ED-2) cells near the portal vessel. Serum chemokine (C-X-C motif) ligand 1 (CXCL-1), CXCL-2 and chemokine (C-C motif) ligand 2 (CCL-2) showed a peak at 3 h after both treatment regimens although at a higher extent after Zymosan administration. Accordingly, CXCL-1, CXCL-5 and CCL-2 gene expression in the liver was up-regulated after GdCl_3_ treatment at 3 h. After Zymosan administration a significant up-regulation of CXCL-1, CXCL-2, CXCL-10, CCL-2, CCL-3 and CCL-20 gene expression in liver at 3 h was observed. After Zymosan administration intracellular adhesion molecule 1 (ICAM-1) and vascular cell adhesion molecule 1 (VCAM-1) gene expression was up-regulated in rat liver tissue. In C3H/HeJ mice both treatment regimens up-regulated CCL-2 and ICAM-1 gene expression after 3 h and down-regulated platelet endothelial cell adhesion molecule 1 (PECAM-1) gene expression. In conclusion, phagocytosis overload of Kupffer cells causes induction of several CXC, CC-chemokines, upregulation of “positive” adhesion molecule gene expression, down-regulation of the “negative” adhesion molecule PECAM-1 and a recruitment of neutrophil granulocytes in the portal area of the liver of treated rats and mice mainly in close contact to the liver macrophages.

## 1. Introduction

The liver is not only an important “power plant” of the body but also one of the main clearance organs. This function is performed mainly by the hepatocyte through the uptake and clearance of xenobiotics and catabolites of heme and by the liver macrophages (Kupffer cells). Liver macrophages represent the bulk of the reticuloendothelial system. This is responsible for the clearance of aged erythrocytes, granulocytes, microorganisms and of other particulate matter as well [1,2].

Kupffer cells exert cellular defence functions for the whole body but also for the liver itself. This function harbors the danger that the substances that should be degraded and/or eliminated lead to cellular damage. Each cell type of the liver, including the hepatocyte, possesses its own defence apparatus consisting mainly of pro-inflammatory chemokines [3]. Liver inflammation and damage in many animal models but also in humans may not be initiated by death (apoptosis or necrosis) of liver parenchymal cells but by liver resident and recruited inflammatory cells [4]. To this purpose resident liver macrophages may communicate with hepatocytes and, possibly, also with inflammatory cells.

Hepatocellular stress, induced by hepatotoxins or viruses, may lead to activation of Kupffer cells on one side and to release of chemokines and/or cytokines on the other side [5,6,7,8,9]. At the same time Kupffer cell blockade by gadolinium chloride (GdCl_3_) treatment could have the consequence of cytokine production by the liver as a response to the phagocytic challenge of GdCl_3_ aggregates and somehow induce the reduction of bile production. In that experimental setting, intravenous injection of gadolinium did not increase serum level of endotoxin [10].

Kupffer cells contribute also to hepatotoxicity via different mechanisms encompassing the formation of nitric oxide and superoxide that react together to produce peroxynitrite, which again exhibits hydroxyl radical-like activity [11]. Liver macrophages, like blood mononuclear phagocytes, also synthesize and secrete cytokines such as interferons, interleukin-1 (IL-1), interleukin-6 (IL-6), tumour necrosis factor-alpha (TNF-α) and chemokines. Injection of GdCl_3_ has been shown to block the phagocytic activity of Kupffer cells induce some phenotypic changes such as the ED-2 disappearance [12]. Previously, inhibition of Kupffer cells has been demonstrated also to protect against hepatic injury such as ischemia/reperfusion injury, alcohol-induced injury, and injuries induced by certain toxicants, such as cycloheximide [13]. Earlier studies reported that GdCl_3_ treatment shortly prior to acetaminophen challenge in mice significantly attenuated liver damage and associated mortality [11,14]. Although intravenously or intraperitoneally injected GdCl_3_ has been used to block the phagocytic capacity of the Kupffer cells, the consequences of this treatment in terms of changes of expression of different hepatic genes has not been studied thoroughly.

Neutrophil granulocytes are the most abundant myeloid leukocytes in mammals. In inflammation scenarios, neutrophil granulocytes recruited to injured tissues are eventually cleared by macrophages, a process that contributes to resolving inflammation and restoring homeostasis [15,16]. Of note, many studies also suggested that neutrophil granulocytes clearance might be a significant source of homeostatic signals able to functionally modulate the tissues and organs where they are eliminated [17,18].

The recruitment of neutrophil granulocytes to the sites of “stress” is preceded by alteration of gene transcription and the modulation of chemokines, pro-inflammatory cytokines and adhesion molecules. An exuberant neutrophil granulocytes recruitment in response to inflammatory stimuli is the consequence and it contributes to the immunopathology observed in many diseases [19,20,21]. In a recent study [2], we could show that a single dose of thioacetamide (TAA) intraperitoneally administrated induces a fast and early increase in CXC-chemokines in the liver, accompanied by an increased number of neutrophil granulocytes around the portal vessel walls; between and around biliary cells and liver myofibroblasts but not in the parenchyma (sinusoids). These data suggested that the production of several chemokines may be necessary to induce recruitment of inflammatory cells beginning around the vessel walls within the portal area. We also demonstrated that immunopathology is sometimes necessary to detect recruitment of neutrophil granulocytes into the portal area after a single dose irradiation, as simple light microscopy was negative [4].

Zymosan is a substance derived from the cell wall of the yeast *Saccharomyces cerevisiae*. It has been extensively used to induce an experimental condition of systemic inflammation [22] and to directly activate in vitro mouse macrophages as a result of phagocytosis [23]. This agent was shown to induce the secretion of IL-8 [24] and TNF-α [25]. Interaction of Zymosan particles with the monocyte β-glucan receptor stimulates rapid phagocytic responses and induces the release of a broad spectrum of biologically active compounds [26]. We have already demonstrated that administration of Zymosan had a comparable effect to that of GdCl_3_ administration. We published quite dramatic changes in gene expression of proteins considered to be involved in hepatic iron metabolism and in hepatic gene expression of acute-phase cytokines, and demonstrated that Kupffer cells are involved in this process [22]. Zymosan is known to trigger an inflammatory response through the activation of Kupffer cells in the liver while the precise effects of GdCl_3_ on Kupffer cells are not still defined.

The current study aims to further extend the understanding of the changes in gene expression of the main CXC- and CC-chemokines as well as of adhesion molecules induced by the uptake of corpuscular matter by the liver macrophages. Moreover, it may be responsible for the recruitment of neutrophil granulocytes causing a possible interaction of the Kupffer cells and granulocytes. To further expand our findings and to rule out any changes caused by endotoxin contamination in our rat model we used C3H/HeJ mice. These mice have a missense mutation in the third exon of toll-like receptor 4 (TLR-4), yielding a non-functional TLR-4; they are hypo-responsive to the effects of lipopolysaccharide and are resistant to lethal endotoxin-induced shock as compared with normal mice.

One of the main objectives of this work, however, is to introduce a caveat for the interpretation of data obtained by injecting different materials intraperitoneally without taking into account the changes in expression of certain genes induced in the liver.

## 2. Results

### 2.1. Immunofluorescence Analysis of the Rat Liver Following Intraperitoneal Injection of GdCl_3_ or Zymosan

**Figure 1:**
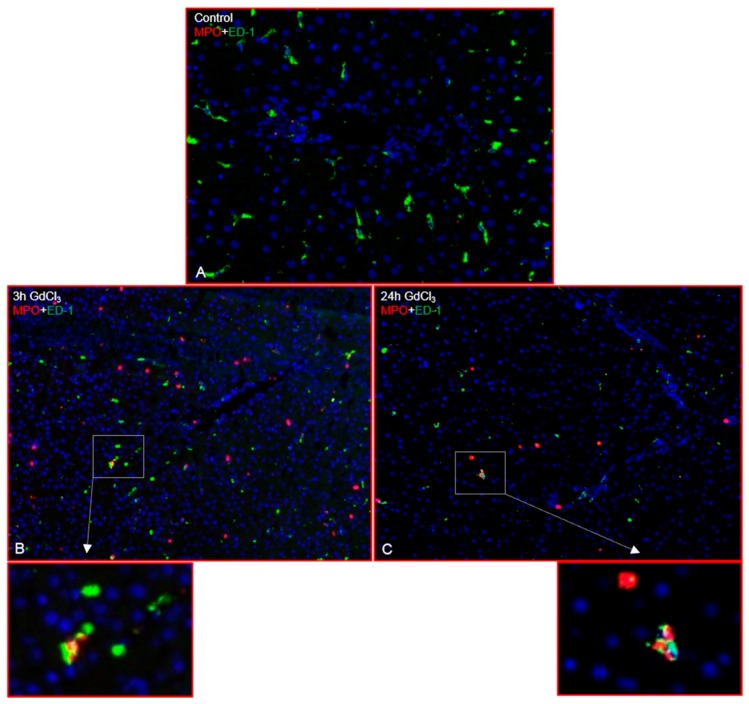
Double immunofluorescence staining of rat liver sections (GdCl_3_ treatment) with antibodies directed against ED-1 (green) and MPO (red) followed by fluorescence immunodetection. Liver sections from different time points of study are shown: control (**A**); 3 h (**B**) and 24 h (**C**). Results shown are representative pictures of six animals and six slides per time point. (Original magnification 200×).

**Figure 2:**
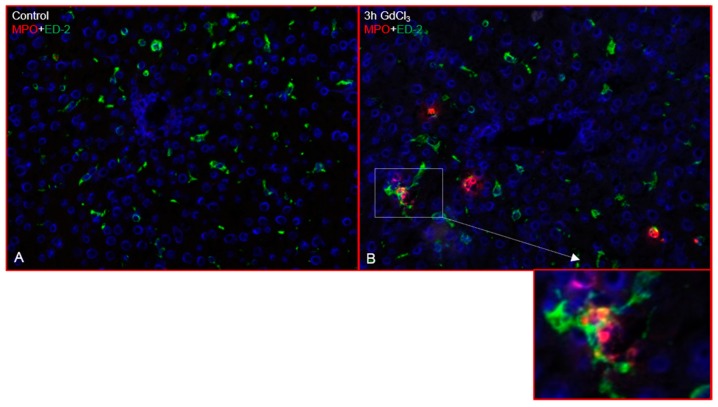
Double immunofluorescence staining of rat liver sections (GdCl_3_ treatment) with antibodies directed against ED-2 (green) and MPO (red) followed by fluorescence immunodetection. Liver sections from different time points of study are shown: control (**A**) and 3 h (**B**). Results shown are representative pictures of six animals and six slides per time point. (Original magnification 200×).

Immunofluorescence double staining showed increased numbers of myeloperoxidase positive (MPO^+^) cells (recruited granulocytes) as early as 3 h (hours) after GdCl_3_ administration (Figure 1B) compared to untreated animals (Figure 1A) while the number of MPO^+^ cells decreased at 24 h after treatment (Figure 1C). MPO^+^ cells were mainly located near the portal vessel and in close vicinity to the liver macrophages. The same liver sections showed a progressive reduction of ED-1 positivity after GdCl_3_ administration, mainly near the portal vessel (Figure 1) while ED-2 positivity remained unchanged (Figure 2). Double immunofluorescence staining showed few ED-1^+^ and MPO^+^, and ED-2^+^ and MPO^+^ cells in close contact to each other near the portal area (Figure 1 and Figure 2).

**Figure 3:**
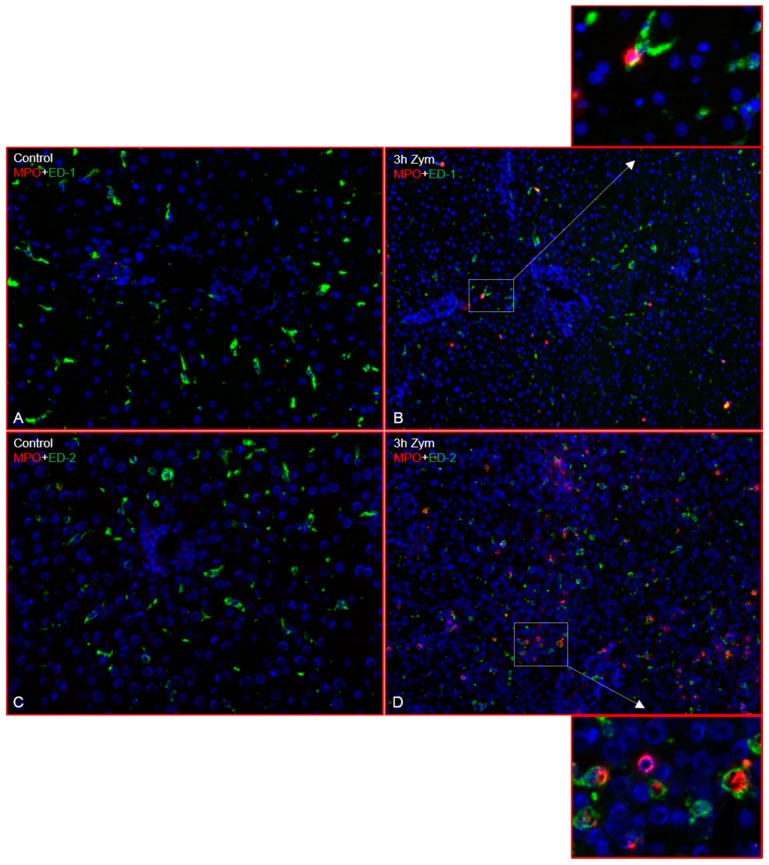
Double immunofluorescence staining of rat liver sections (Zymosan treatment) with antibodies directed against ED-1 and ED-2 (green) and MPO (red) followed by fluorescence immunodetection. Liver sections from different time points of study are shown: control (**A**) and 3 h (**B**) with antibodies against ED-1 and MPO; control (**C**) and 3 h (**D**) with antibodies against ED-2 and MPO. Results shown are representative pictures of six animals and six slides per time point. (Original magnification 200×).

Double immunofluorescence staining of liver sections after Zymosan treatment with antibodies against ED-1, ED-2 and MPO showed an increased number of MPO^+^ cells at 3 h (Figure 3B,D), which were located near the portal vessels but also through the liver parenchyma, compared to controls (Figure 3A). Double immunofluorescence staining showed few MPO^+^/ED-1^+^ as well as MPO^+^/ED-2^+^ cells near the portal area (Figure 3A–D).

### 2.2. Changes in the Serum Levels of CXCL-1, CXCL-2 and CCL-2 of Rat after GdCl_3_ or Zymosan Administration

**Figure 4:**
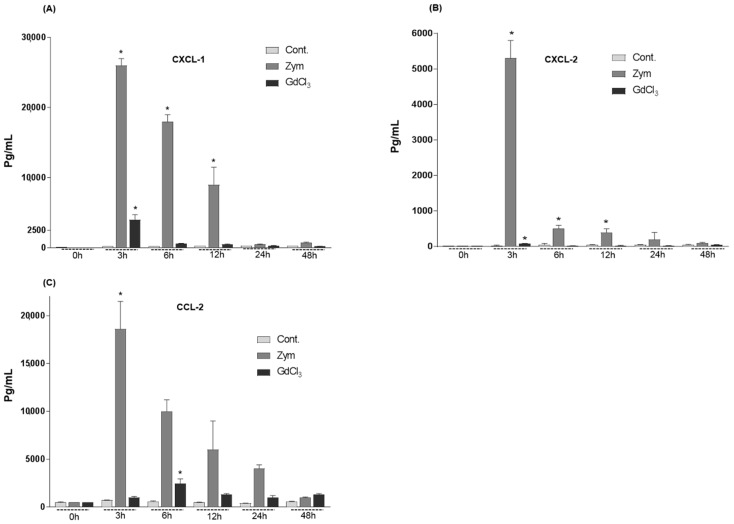
Changes in the serum levels of CXCL-1, CXCL-2 and CCL-2 in intraperitoneally administrated GdCl_3_ or Zymosan rats by enzyme-linked immunosorbent assay (ELISA) measurement. (**A**) CXCL-1 levels in the serum of intraperitoneally administrated GdCl_3_ or Zymosan rats. Zymosan treated rats shows pronounced CXCL-1 serum levels at 3 h than GdCl_3_ treated rats. (**B**) CXCL-2 levels in the serum of GdCl_3_ and Zymosan treated rats. Zymosan treated rats shows pronounced CXCL-2 serum levels at 3 h than GdCl_3_ treated rats. (**C**) CCL-2 levels in the serum of GdCl_3_ and Zymosan treated rats. Zymosan treated rats shows pronounced CCL-2 serum levels at 3 h, while in GdCl_3_ treated rats increased slightly at 6 h. Values on the y-axis show the serum concentration of CXCL-1, CXCL-2 and CCL2 measured with ELISA. These results are representative of six animal series (* *p* < 0.05; mean ± standard error of the mean (SEM)).

Changes in the level of CXCL-1, CXCL-2 and of CCL-2 measured by enzyme-linked immunosorbent assay (ELISA) at different time points in sera after intraperitoneal administration of GdCl_3_ and Zymosan were compared with values obtained in control rats. In GdCl_3_ administrated rats, a time-dependent significant increase of serum CXCL-1 (at 3 h, 3970 ± 750 pg/mL), CXCL-2 (at 3 h, 80 ± 10 pg/mL and of CCL-2 (at 6 h, 2460 ± 490 pg/mL) was detected. Zymosan administration induced a sharp increase of CXCL-1 (26,000 ± 1000 pg/mL), CXCL-2 (5300 ± 500 pg/mL) and of CCL-2 (18,600 ± 2900 pg/mL) serum levels reaching a peak at 3 h (Figure 4A–C). Although a clear decrease of serum level of the measured chemokines was continuously observed thereafter, normal serum levels were not definitively reached even at 48 h after injection of Zymosan, while only CXCL-1 reversed to control levels at 48 h after GdCl_3_ administration (Figure 4A).

### 2.3. Expression of CXC- and CC-Chemokines in Rat Livers after GdCl_3_ Administration

**Figure 5:**
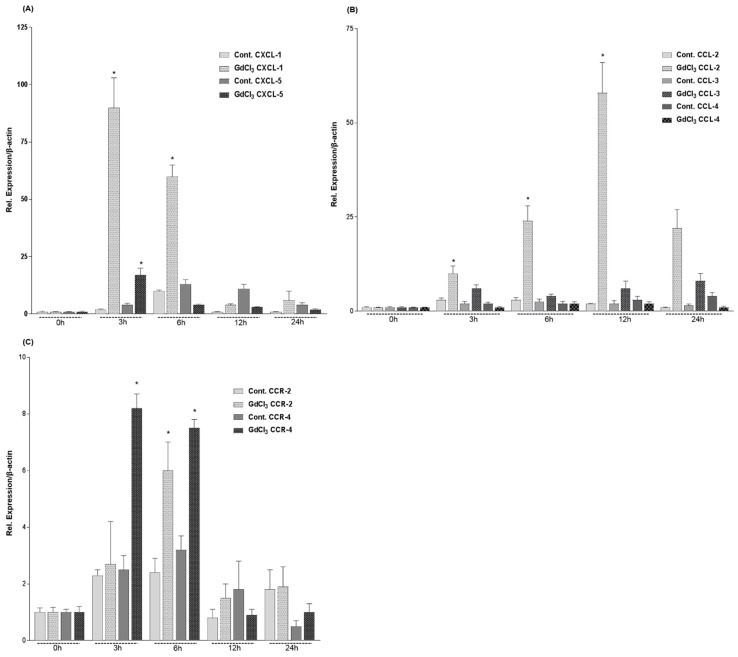
Fold change of mRNA expression of CXC-, CC-chemokines and CC-receptors in intraperitoneally administrated GdCl_3_ rat livers at different time points relative to controls. (**A**) Maximum increase of CXCL-1 and CXCL-5 gene expression is detected at 3 h by real-time polymerase chain reaction (RT-PCR). (**B**) CCL-2 gene expression slightly increased at 3 h and 6 h with a maximum at 12 h by RT-PCR, whereas CCL-3 and CCL-4 did not show any increase. (**C**) CCR-2 gene expression significantly increased at 6 h by RT-PCR whereas CCR-4 showed a significant increase already at 3 h. The results were normalized to the housekeeping gene β-actin and experimental errors are shown as ±SEM values of six experiments (in duplicate) compared with controls for each time point (* *p* < 0.05, analyzed by one-way analysis of variance (ANOVA)).

Real-time polymerase chain reaction (RT-PCR) analysis of total RNA extracted from livers of GdCl_3_ administrated rats (Figure 5) showed a significant up-regulation of the CXC-chemokines CXCL-1 (90 ± 13 fold) and of CXCL-5 (17 ± 3 fold) that reached a peak at 3 h (Figure 5A). The intraperitoneal administration of GdCl_3_ also induced an up-regulation in the gene expression of CC-chemokines; 58 ± 8 fold increase in CCL-2 mRNA levels at 12 h (Figure 5B), a 6 ± 1 fold increase of CCR-2 mRNA level at 6 h and 8 ± 0.5 fold increase of CCR-4 mRNA level at 3 h (Figure 5C).

### 2.4. Expression of CXC-, CC-Chemokines and Chemokine Receptors in Rat Liver after Zymosan Administration

**Figure 6:**
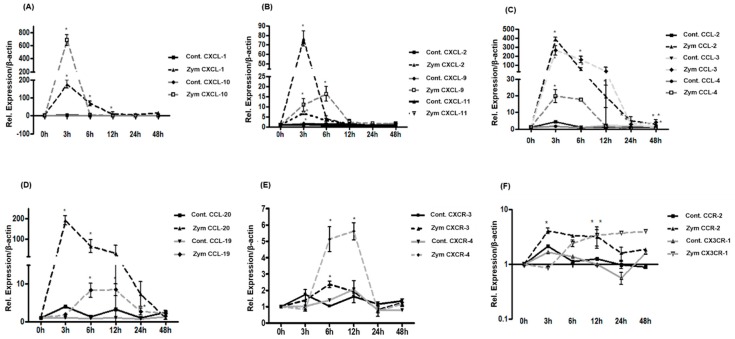
Fold change of mRNA expression of CXC-, CC-chemokines, CC- and CXC-chemokine receptors in intraperitoneally administrated Zymosan rat livers at different time points relative to controls. (**A**,**B**) Maximum increase of CXCL-1, CXCL-2, CXCL-10 and CXCL-11 gene expression is detected at 3 h by RT-PCR, whereas the maximum gene expression of CXCL-9 is detected at 6 h. (**C**,**D**) Maximum increase of CCL-2, CCL-3, CCL-4 and CCL-20 gene expression is detected at 3 h by RT-PCR and only CCL-19 gene expression increased significantly at 6 h. (**E**) CXCR-3 gene expression significantly increased at 6 h by RT-PCR whereas CXCR-4 showed a significant increase already at 12 h. The results were normalized to the housekeeping gene *β-actin* and experimental errors are shown as ±SEM values of six experiments (in duplicate) compared with controls for each time point (* *p* < 0.05, analyzed by one-way ANOVA). (**F**) CCR-2 gene expression significantly increased at 6 h by RT-PCR whereas CX3CR-1 showed no significant increase. The results were normalized to the housekeeping gene β-actin and experimental errors are shown as ±SEM values of six experiments (in duplicate) compared with controls for each time point (* *p* < 0.05, analyzed by one-way ANOVA).

RT-PCR analysis of total RNA extracted from Zymosan administrated rat livers showed an up-regulation of CXCL-1 (175 ± 21 fold) and CXCL-10 (681 ± 61 fold) gene expression with a maximum at 3 h (Figure 6A). The gene expression of CXCL-2 (76 ± 8 fold) and CXCL-11 (7 ± 0.9 fold) also increased reaching a peak level at 3 h after Zymosan administration (Figure 6B). At the same time the gene expression of CXCL-9 was significantly up-regulated (16 ± 3 fold) at 6 h after Zymosan injection (Figure 6B) compared to the controls. Moreover, the gene expression of several CC-chemokines such as CCL-2 (390 ± 20 fold), CCL-3 (265 ± 40 fold) and CCL-20 (190 ± 22 fold) was dramatically increased by 3 h of treatment (Figure 6C,D) and returned gradually to normal level. Quantitative real-time PCR also revealed a statistically significant up-regulation of CCL-4 (19 ± 3 fold) and CCL-19 (8 ± 1 fold) reaching the peak by 3 h and 12 h (Figure 6C,D). Analysis of CC- and CXC-chemokine receptors showed an up-regulation of CXCR-3 and CXCR-4 gene expression to a maximum level of 5 ± 1 fold by 12 h and 2.4 ± 1 fold by 6 h respectively, after Zymosan injection (Figure 6E) whereas no significant changes at mRNA level for the CC-receptors were detected (Figure 6F).

### 2.5. Expression of the Genes of Adhesion Molecules in Rat Livers after GdCl_3_ or Zymosan Administration

**Figure 7:**
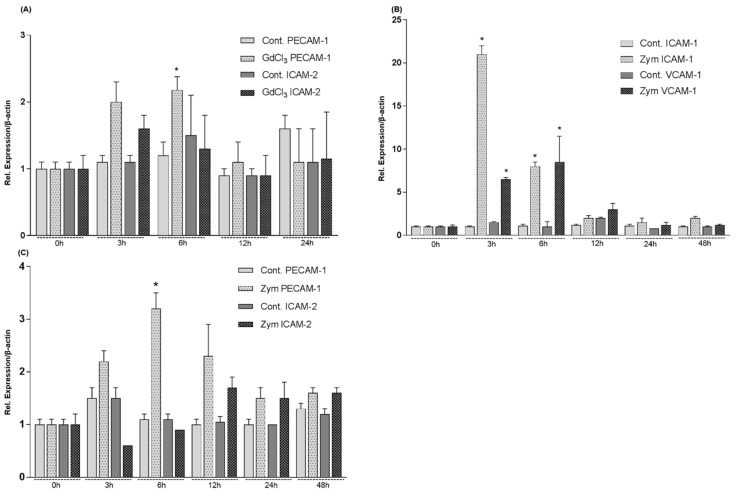
Fold change of mRNA expression of adhesion molecules in intraperitoneally administrated GdCl_3_ and Zymosan rat livers at different time points related to controls. (**A**) Maximum increase of PECAM-1 gene expression is detected at 6 h by RT-PCR and of ICAM-2 at 3 h in GdCl_3_ treated rat livers. (**B**) Maximum increase of ICAM-1 gene expression is detected at 3 h by RT-PCR whereas VCAM-1 gene expression showed a significant increase at 6 h. (**C**): Maximum increase of PECAM-1 gene expression is detected at 6 h by RT-PCR, whereas ICAM-2 gene expression increased at 12 h in Zymosan treated rat livers. The results were normalized to the housekeeping gene β-actin and experimental errors are shown as ±SEM values of six experiments (in duplicate) compared with controls for each time point (* *p* < 0.05, analyzed by one-way ANOVA).

Hepatic expression of the intracellular adhesion molecule 1 (ICAM-1), intracellular adhesion molecule 2 (ICAM-2), platelet endothelial cell adhesion molecule 1 (PECAM-1) and vascular cell adhesion molecule 1 (VCAM-1) was analyzed by quantitative RT-PCR in rat models of intraperitoneally administrated GdCl_3_ or Zymosan (Figure 7). GdCl_3_ stimulated VCAM-1 gene expression, although the results were not statistically significant (data not shown), and PECAM-1 reaching the highest level of 2.1 ± 0.2 fold at 6 h (Figure 7A). Zymosan induced a sharp increase of ICAM-1 and VCAM-1 which reached the highest level after 3 h (21 ± 1 fold) and 6 h (8.5 ± 3 fold) respectively, and induced PECAM-1 gene expression 3.2 ± 0.3 fold at 6 h after injection whereas ICAM-2 was not significantly regulated (Figure 7B,C).

### 2.6. Expression of CC- and CXC-Chemokine Receptors in C3H/HeJ Mice after GdCl_3_ or Zymosan Administration

**Figure 8:**
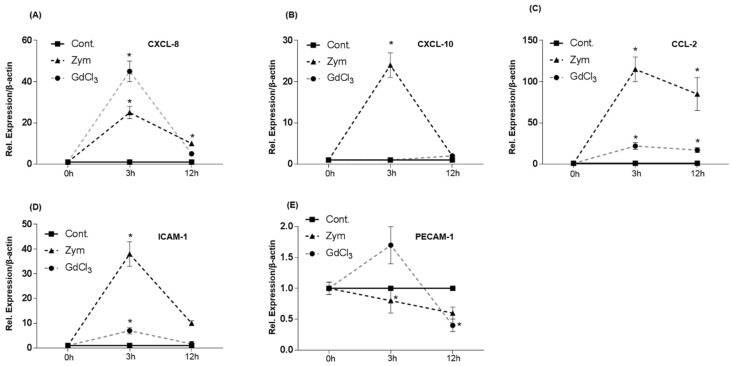
Fold change of mRNA expression of CXC-, CC-chemokines and adhesion molecules in intraperitoneally administrated GdCl_3_ and Zymosan C3H/HeJ mice livers at different time points relative to controls. Maximum increase of CXCL-8 (**A**), CXCL-10 (**B**) and CCL-2 (**C**) gene expression is detected at 3 h by RT-PCR. Maximum increase of ICAM-1 (**D**) and PECAM-1 (**E**) gene expression is detected at 3 h by RT-PCR in Zymosan treated mice livers. The results were normalized to the housekeeping gene β-actin and experimental errors are shown as ±SEM values of six experiments (in duplicate) compared with controls for each time point (* *p* < 0.05, analyzed by one-way ANOVA).

To rule out a role for endotoxin contamination in the results observed in GdCl_3_ and Zymosan treated rats, C3H/HeJ endotoxin-resistant mice were treated with GdCl_3_ or Zymosan administered intraperitoneally. Zymosan induced an up-regulation of CXCL-8 (28 ± 5 fold), CXCL-10 (24 ± 3 fold) and CCL-2 (115 ± 15 fold) gene expression at 3 h after treatment (Figure 8A–C). GdCl_3_ induced a sharp up-regulation of CCL-2 (22 ± 4 fold) gene expression after 3 h of administration, whereas CXCL-10 up-regulation was not significantly changed (Figure 8B,C).

Of note, GdCl_3_ and Zymosan intraperitoneal treatment significantly up-regulated ICAM-1 gene expression at 3 h (7 ± 1.3 fold and 38 ± 5 fold respectively, Figure 8D) whereas both stimulants slightly down-regulated PECAM-1 gene expression (Figure 8E).

## 3. Discussion

Acute and chronic liver injuries are accompanied by a prominent inflammatory response including an increased expression of CXC- and CC-chemokines and their receptors. Release of some chemo-attractants from hepatocytes and liver macrophages (Kupffer cells) “stressed” by toxic agents such as carbon tetrachloride is considered to be responsible for recruitment of inflammatory cells but little is still known about the changes in the expression of pro-inflammatory chemokines and adhesion molecules during activation by “phagocytic” overload of Kupffer cells. Hardonk et al. demonstrated the specific effect of GdCl_3_ in the liver as large Kupffer cells disappeared after treatment whereas white pulp spleen macrophages did not change after GdCl_3_ administration [27].

The results of this study demonstrate, at transcriptional and translational level, that activation by overload of Kupffer cells leads to increase of specific mRNA and specific proteins of the chemokines involved in recruitment of Neutrophil granulocytes in the portal area of the liver. Up-regulation of the main pro-inflammatory chemokines was concomitant with that of the main adhesion molecules in the liver. This in vivo animal model demonstrates the qualitative changes of Kupffer cells and the increased number of neutrophil granulocytes after activation by overload of Kupffer cells mainly at 3 h. At the same time point gene expression of CXCL-2 and CXCL-10 as well as of CCL-2, CCL-3, CCL-4 and of CCL-20 were strongly up-regulated in the liver after intraperitoneal administration of Zymosan. Intraperitoneal injection of GdCl_3_ stimulated the gene expression of CXCL-1, CXCL-2, CXCL-5, and CCL-2 reaching a maximum level by 3 h and 12 h after treatment. However, the magnitude of gene expression induction of chemokines by Zymosan was stronger than GdCl_3_. CXCL-10 mRNA was the most induced following Zymosan administration. One could speculate that the changes of the gene expression of these chemokines are responsible for the recruitment of the neutrophil granulocytes. While increase of the gene expression of the main acute-phase cytokines in liver tissue and in the serum most probably takes place in the overloaded Kupffer cells, which cell(s) is mainly responsible for the production of the chemokines measured in this work remains to be elucidated. Several studies could show that parenchymal and non-parenchymal cells of the liver are able to produce these chemokines [3,9,28].

The magnitude of chemokines mRNA expression after GdCl_3_ or Zymosan administration was consistent to that observed after other acutely induced liver damage models, such as carbon tetrachloride administration [29] or irradiation of the rat liver [30]. When serum concentrations of the chemokines CXCL-1, CXCL-2 and CCL-2 after GdCl_3_ or Zymosan administration were measured, we could detect an abundant release of the chemokines induced by Zymosan and to a lesser extent by GdCl_3_, reaching a nadir 3 h after injection. Thus, it is noticeable that CXCL-1 was detected at the highest serum concentration levels after intraperitoneal administration of GdCl_3_ or Zymosan. The increase of CXCL-1, CXCL-2 and CCL-2 serum levels could be related to rapid recruitment of neutrophil granulocytes from the blood. On the other side, the intraperitoneal injection of both agents used in this study could induce a dramatic local production of pro-inflammatory molecules within the peritoneum. This consideration must, however, be tempered by the correlation of chemokine serum measurement and mRNA levels in the liver observed after GdCl_3_ and Zymosan injection. Furthermore, the finding of some granulocytes closely attached to Kupffer cells allows the conclusion that changes in gene expression of both chemokine and adhesion molecule take place in the overloaded Kupffer cells. In some cases, neutrophil granulocytes seemed to be “contained” within Kupffer cells.

Moreover, Moriconi et al. [30] and Malik et al. [26] have shown that liver irradiation induces an up-regulation of the main pro-inflammatory chemokines in a rat model of liver irradiation, such as CXCL-1/CINC1, CXCL-10, CCL-2 and CCL-20 without the massive recruitment of leukocytes into the liver parenchyma that one would expect [31]. A reason for this finding could be that local chemokine up-regulation alone is not sufficient to induce leukocyte transmigration suggesting that other mechanisms, such as a stronger modulation of gene expression of the main adhesion molecules [30], could play a decisive role in this process. The extravasation of inflammatory cells from the blood into the “stressed” tissue is mediated by changes of gene expression of adhesion molecules on the inflammatory blood cells, which mediate the attachment to the cells of the sinusoidal lumen such as Kupffer cells and transmigration of activated leukocytes toward the endothelium before reaching the hepatocytes. The induction of such changes in the cells flowing through the hepatic sinusoid may need a stronger signal than that induced by a single dose of GdCl_3_ or Zymosan administration.

PECAM-1 belongs to the immunoglobulin’s superfamily of cell adhesion molecules; it has been shown to activate a variety of pro-inflammatory and anti-inflammatory functions in both leukocytes and endothelial cells [32]. However, the specific role of PECAM-1 during inflammation is controversial. Previously we could demonstrate [33] that PECAM-1 gene expression is down-regulated during in vitro activation of peripheral blood leukocytes in parallel with an increase in ICAM-1 and VCAM-1 expression, a prerequisite for transmigration of inflammatory leukocytes into tissue. In that previous study we were also able to identify one possible mechanism responsible for PECAM-1 blockage as an anti-TNF-α antibody exerted its anti-inflammatory effect preventing PECAM-1 down-regulation and up-regulation of VCAM-1 and ICAM-1 on blood leukocytes as well as down-regulating IFN-γ gene expression.

The changes in gene expression of the main pro-inflammatory chemokines occurred in the absence of hepatotoxicity, as serum AST and ALT levels did not increase in these experiments. The present data demonstrate that activation and overload of Kupffer cells regulate the gene expression of the main pro-inflammatory chemokines in the liver of both rats and mice without abundant recruitment of Neutrophil granulocytes into the liver parenchyma. A possible explanation for these findings could be the local down-regulation of PECAM-1 gene expression after GdCl_3_ or Zymosan intraperitoneal injection, which we found to be down-regulated under “inflammatory” conditions [33]. 

To rule out the possibility that the observed effect could be due to endotoxin contamination, endotoxin-resistant (C3H/HeJ) mice, which are hypo-responsive to the effects of lipopolysaccharide [34,35], were also studied. GdCl_3_ and Zymosan induced an up-regulation of CCL-2 and ICAM-1 gene expression in C3H/HeJ mice. As a consequence, the findings in a rat model were not an endotoxin contamination of the injected substances. In contrast, GdCl_3_ and Zymosan treatment in C3H/HeJ mice induced a slight down-regulation of PECAM-1 together with an up-regulation of ICAM-1 gene expression.

These results further highlight the complexities of chemokine interactions and their contribution to regulating neutrophil granulocytes recruitment in liver tissue of different animal species [3,36,37]. The present data also highlight that a mild recruitment of neutrophil granulocytes in the liver parenchyma soon after GdCl_3_ and Zymosan administration is taking place without a detectable tissue damage and that the recruited cells may be, at least in part, eliminated by phagocytosis. Neutrophil granulocytes, after having delivered the load of proteases, can, however, be transported by the sinusoidal blood and taken up by tissue macrophages in other organs [38].

This data also adds support to the suggestion that “overloading” of the phagocytic capacity may induce significant pro-inflammatory changes of liver gene expression as previously shown for the proteins involved in iron metabolism in the liver [2]. Taken together, data of our present study showed that GdCl_3_ administration itself could be involved in triggering hepatic inflammatory response, albeit to a lesser extent compared to the hepatic inflammatory response induced by Zymosan.

## 4. Materials and Methods

### 4.1. Animals

Male Wistar rats (8 weeks old) and C3H/HeJ mice were purchased from Charles River (Sulzfeld, Germany). They were maintained under standard conditions with 12 h light and dark cycles and allowed ad libitum access to fresh water and food pellets consistent with the university’s policies and guidelines for the care and use of laboratory animals. The use of rat in this research was reviewed, approved, and overseen by the local ethics committee of the University of Goettingen as well as the public authority on animal welfare.

### 4.2. Reagents

All the chemicals used were of analytical grade and purchased from commercial sources as indicated: Real-time PCR primers from Invitrogen (Carlsbad, CA, USA), M-MLV reverse transcriptase, reverse transcription buffer and 0.1 M DTT, platinum Sybr green qPCR-UDG mix from Invitrogen (Groningen, The Netherlands), dNTPs, protector RNase inhibitor, primer oligo(DT)_15_ for cDNA synthesis and salmon sperm DNA from Roche (Mannheim, Germany).

### 4.3. Antibodies

ED-1 (mouse monoclonal antibody, MCA 341R, Serotec, Puchheim, Germany) and ED-2 (mouse monoclonal antibody, MCA 342R, Serotec). A rabbit polyclonal antibody against myeloperoxidase (MPO) was purchased from Dako (Hamburg, Germany). Fluorescent labelled antibodies Alexa Fluor 555 goat anti-rabbit or Alexa Fluor 488 goat anti-mouse were purchased from Invitrogen (Darmstadt, Germany).

### 4.4. GdCl_3_ and Zymosan Administration to Rats and C3H/HeJ Endotoxin-Resistant Mice

One experimental group of rats was injected intraperitoneally with single dose of sterile and pyrogen-free GdCl_3_ solution (40 mg/Kg of body) and other group received an injection of sterile pyrogen-free Zymosan (100 mg/Kg of body). While the control group of rats was injected with sterile saline solution intraperitoneally. To further rule out a role for endotoxin contamination in the results observed in GdCl_3_ and Zymosan-administrated rats, C3H/HeJ endotoxin-resistant mice were administrated GdCl_3_ or Zymosan intraperitoneally. Animals of all experimental groups were killed under pentobarbital anesthesia. Livers were rinsed and snap-frozen in liquid nitrogen. Samples were stored at −80 °C until further use. All experiments were repeated independently in six series. From each experimental group, animals were killed at different time points of the study.

### 4.5. Liver Immunohistochemistry

The immunofluorescence studies were performed according to a protocol described previously [39]. Five micrometer cryostat sections of rat livers were cut, air-dried and fixed with acetone (−20 °C for 10 min). After fixation, tissue sections were blocked with 1% bovine serum albumin and 10% goat serum (1 h at room temperature) in a humidified chamber to inhibit non-specific binding of the antibody. In the next step after blocking, we washed the tissue sections with phosphate-buffered saline (PBS) three times for 10 min each. Thereafter, sections were incubated with primary antibody overnight at +4 °C. After overnight incubation, the slides were again washed three times in PBS, followed by incubation with fluorescence-labelled secondary antibody: Alexa Fluor 555 goat anti-rabbit or Alexa Fluor 488 goat anti-mouse at room temperature for 1 h in darkness. The counterstaining of the nuclei was done with diamidino-2-phenylindole (DAPI) (4 µL in 100 mL of PBS). The slides were mounted with Fluoromount-G and observed with an epifluorescence microscope (Axiovert 200M; Zeiss, Jena, Germany).

### 4.6. RNA Isolation and Quantitative Real-Time Polymerase Chain Reaction (PCR)

Total RNA from the rat liver tissue samples was isolated with Trizol reagent according to the manufacturer’s instructions. The RNA was then quantified by measuring the absorbance at 260/280 nm. For real-time PCR, reverse transcription of RNA samples was performed using the Superscript kit from Invitrogen (Groningen, The Netherlands) and following the manufacturer’s instructions the cDNA was synthesized as previously described [40]. RT-PCR of cDNA was performed at 95-60 °C for 45 cycles in the Sequence Detection System of ABI Prism 7000 (Applied Biosystems, Darmstadt, Germany) following the manufacturer’s instructions and using SYBR Green Reaction Master Mix (ABI Prism) and the primers listed in Table 1. In every RNA sample, β-actin mRNA is measured as the housekeeping gene (ubiquitin C was also measured showing very similar results). Values were then compared with those obtained using the control-RNA of non-treated rats. The results were normalized to the housekeeping gene and fold change expression was calculated using threshold cycle (Ct) values.

### 4.7. Enzyme-Linked Immunosorbent Assay (ELISA)

For detection of CXCL-1 (R&D Systems, Wiesbaden, Germany), CXCL-2 and CCL-2 (Biosource, Solingen, Germany) in serum of rats administrated with GdCl_3_ and Zymosan, an ELISA kit was used. Samples were processed according to the manufacturer’s instructions.

### 4.8. Statistical Analysis

The data were analysed using GraphPad Prism 5 software (San Diego, CA, USA). Data are presented as mean and standard error of the mean (SEM). A student’s *t*-test was carried out to calculate significant differences among treated (GdCl_3_ and Zymosan) and control data groups. Statistically significant differences, compared with the control groups were estimated also by one-way ANOVA. For calculation of relative changes, the gene expression before treatment was set as “1”. Differences with a *p*-value of ≤0.05 after adjusting for multiple comparisons were considered significant.

## Figures and Tables

**Table 1:** Sequence of gene-specific primers used for quantitative RT-PCR analysis.

Primer	Forward 5′ → 3′	Reverse 5′ → 3′
CXCL-1	GGC AGG GAT TCA CTT CAA GA	GCC ATC GGT GCA ATC TAT CT
CXCL-2	ATC CAG AGC TTG ACG GTG AC	AGG TAC GAT CCA GGC TTC CT
CXCL-5	CTC AAG CTG CTC CTT TCT CG	GCG ATC ATT TTG GGG TTA AT
CXCL-9	GCC TTG ACT CCA GCA CGG T	GAC TTC ATG GCA GAG CCG AG
CXCL-10	CTG TCG TTC TCT GCC TCG TG	GGA TCC CTT CTT GAG TCC CAC TCA
CXCL-11	AGA ACA TGT GAT GGG CCC TC	GGG TCA GCT TCT TGG CAC AG
CCL-2	AGG CAG ATG CAG TTA ATG CCC	ACA CCT GCT GCT GGT GAT TCT C
CCL-3	TTT TGA GAC CAG CAG CAG CCT TT	CTC AAG CCC CTG CTC TAC AC
CCL-4	TCC CGG AAG ATT CAT CGG	GCA CAG ATT TGC CTG CCT TTT
CCL-19	AGA ACG CAT CAT CCG AAG AC	TGC TCA CAC TCA CGT TCA CA
CCL-20	CAA CTT TGA CTG CTG CCT CA	TTC CAT CCC AGA AAA GCA TC
CCR-2	CTT GTG GCC CTT ATT TTC CA	AGA TGA GCC TCA CAG CCC TA
CCR-4	GAA TGC CAC AGA TGT CAC AG	GCA CAA ACA GTA AAT CCG AG
CXCR-3	TAC CTT GAG GTC AGT GAA CG	AAA GAG GAG GCT GTA GAG GA
CXCR-4	GCT GAG GAG CAT GAC AGA CA	GAT GAA GGC CAG GAT GAG AA
CX3CR-1	TGA CTG GCA GAT CCA GAG GTT	GTA GAA TAT GGA CAG GAA CAC
ICAM-1	TGC ACG TCC CTG GTG ATA CTC	TGT CAA ACG GGA GAT GAA TGG
ICAM-2	AGC AGC AGG CAG AGA GTT TC	TCT GCC ACA GAG CAG AGA GA
PECAM-1	TCA GCT GCC AGT CAG TAA ATG G	TCT GGA AGT TGC TCT TTG CTC TT
VCAM-1	ACA TGT GCT GCT GTT GGC TGT	GCT CAG CGT CAG TGT GGA TGT A
β-actin	ACC ACC ATG TAG CCA GGC ATT	CCA CAC AGA GTA CTT GCC CTC A
